# Anti-synthetase syndrome-associated interstitial lung disease possibly caused by atezolizumab in a patient with lung adenocarcinoma: a case report

**DOI:** 10.1186/s12890-023-02446-z

**Published:** 2023-05-08

**Authors:** Ippei Miyamoto, Tetsuo Shimizu, Ryo Kusahana, Masayuki Nomoto, Daishi Fujiwara, Tsukasa Nishizawa, Kentaro Hayashi, Yoshiko Nakagawa, Yasuhiro Gon

**Affiliations:** Department of Respiratory Medicine, Nihon University Itabashi Hospital, Nihon University School of Medicine, 30-1, Oyaguchi-Kamicho, Itabashi-Ku, Tokyo, 173-8610 Japan

**Keywords:** Aminoacyl-tRNA synthetase (ARS), Anti-synthetase syndrome (ASS), Interstitial lung disease, Lung cancer, Immune checkpoint inhibitors (ICIs)

## Abstract

**Background:**

Anti-aminoacyl-tRNA synthetase (ARS) antibody-positive patients present with a variety of symptoms, including interstitial lung disease (ILD), which is termed anti-synthetase syndrome (ASS). But it is rare that ASS-ILD is considered an immune-related adverse event after the use of immune checkpoint inhibitors (ICIs).

**Case presentation:**

A 47-year-old male with advanced lung adenocarcinoma was treated with platinum and ICI combination immunotherapy and was followed up as an outpatient. Nine months after the start of treatment, he developed a fever and cough, and imaging findings showed lung consolidations in the bilateral lower lung fields. The patient was positive for anti- ARS antibodies and was considered to have developed ASS-ILD due to ICIs remitted with steroid therapy. The patient was found to be positive for anti-ARS antibodies before ICI administration, and the antibody titer was elevated compared to that before ICI administration.

**Conclusions:**

The examination of anti-ARS antibodies pior to the administration of ICIs may be useful in predicting the development of ASS-ILD.

## Background

Anti-aminoacyl-tRNA synthetase (ARS) antibodies are autoantibodies frequently found in polymyositis and dermatomyositis. Ten types of anti-ARS antibodies have been identified to date [1, 2]. Anti-ARS antibody-positive patients present with a variety of symptoms, including myositis, interstitial lung disease (ILD), and fever, which is termed anti-synthetase syndrome (ASS) [3]. In ASS, only pulmonary involvement precedes the disease in many cases, and ASS-ILD often predominates, especially in anti-PL-12 antibody-positive patients [4, 5]. Immune checkpoint inhibitors (ICIs) have been increasingly used in the treatment of lung cancer in recent years and are expected to improve prognosis. Conversely, ICIs may induce systemic immune-related adverse events (irAEs) due to immune activation [6], including the development of autoimmune diseases. Although there have been some cases of interstitial pneumonia after ICIs in patients with known ASS and cases of dermatomyositis, lung cancer, or interstitial pneumonia at the time of initial diagnosis [7, 8], it is rare that ASS-ILD is considered an irAE after the use of ICIs.

## Case presentation

A 47-year-old male with advanced lung adenocarcinoma was treated with carboplatin (area under the blood concentration–time curve (AUC) 6 on Day 1) + nab-paclitaxel (nab-PTX 100 mg/m^2^ on Days 1, 8 and 15 of a 21-day cycle) + atezolizumab (1200 mg on Day 1) for a total of 4 courses followed by atezolizumab maintenance therapy. He had clinical T4N3M1b stage IVA lung cancer and was negative for all driver gene mutations (EGFR-, ALK-, ROS1-), and programmed death-ligand 1 (PD-L1) tumor proportion score was expressed at low levels. He had a 26 pack-year history of smoking but had quit smoking before the start of treatment and had no other preexisting medical conditions except lung cancer. During the course of maintenance therapy with atezolizumab, he developed a fever of 38 °C and cough and came to our hospital. He had been treated with atezolizumab for 10 courses, and it had been 9 months since the first dose. SpO_2_ was 94% (room air) at the time of admission, and a slight decrease in SpO_2_ was observed.

**Fig. 1:**
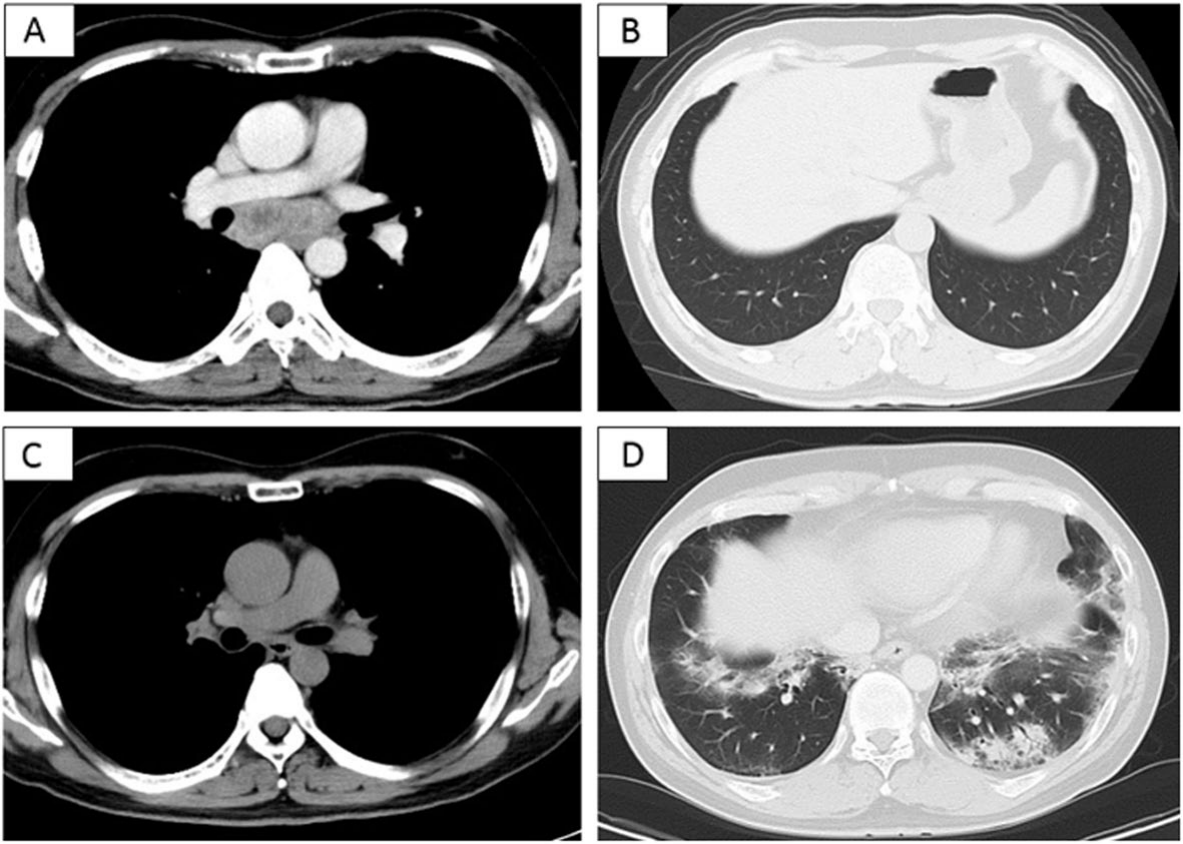
Progress of chest computed tomography (CT) images: **A**-**B** Chest CT findings before atezolizumab administration. **C**-**D** Chest CT findings on admission. Reduction of mediastinal lymph nodes was observed, but peribronchial consolidation appeared

Chest X-ray showed lung consolidation and decreased lung volume in the bilateral lower lung fields. Chest computed tomography (CT) showed that the lymph node metastases in the sub-bifurcation of the tracheal bifurcation were significantly reduced compared with those before the start of chemotherapy, indicating a partial response (PR) to chemotherapy. However, volume reduction in the lower lobes of both lungs and peribronchial consolidation were observed (Fig. 1). The patient was admitted to the hospital after the discontinuation of atezolizumab because of drug-induced interstitial pneumonia, grade 2 on the Common Terminology Criteria for Adverse Events (CTCAE) version 5.0.

**Fig. 2:**
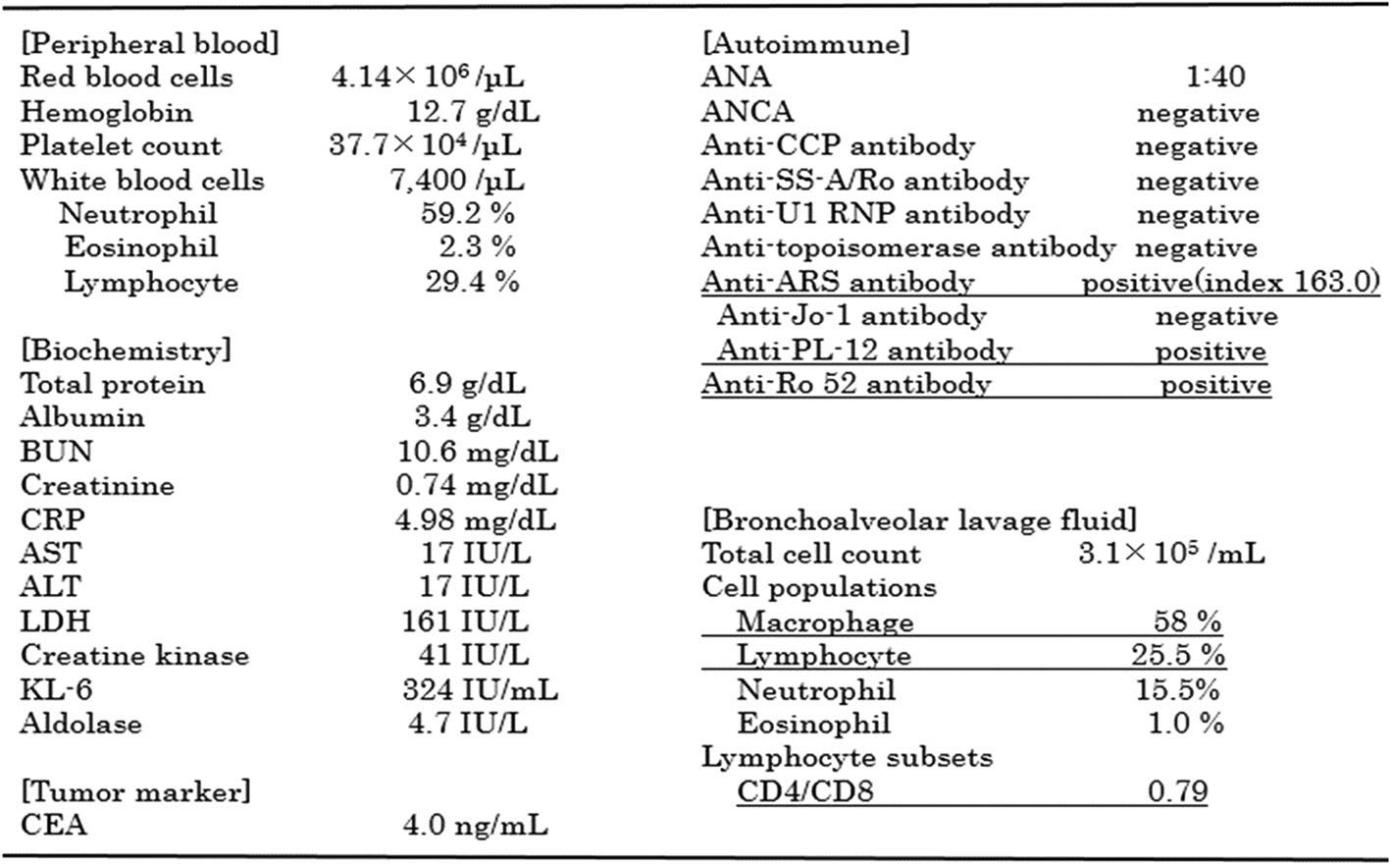
Results of the laboratory investigations after admission. BUN: blood urea nitrogen, CRP: C reactive protein, AST: aspartate aminotransferase, ALT: alanine aminotransferase, LDH: lactate dehydrogenase, KL-6: Krebs von den Lungen-6, CEA: carcinoembryonic antigen, ANA: antinuclear antibody, ANCA: antineutrophil cytoplasmic antibody, Anti-CCP antibody: anti cyclic citrullinated peptides antibody, Anti-SS-A/Ro antibody: anti Sjogren syndrome-A/Ro antibody, Anti-U1 RNP antibody: Anti-U1 ribonucleoprotein antibody, Anti-PL12 antibody: anti-alanyl-tRNA synthetase antibody, CD4: cluster of differentiation 4, CD8: cluster of differentiation 8

After admission, bronchoscopy was performed, and bronchoalveolar lavage (BAL) was performed on the left B^5^. The recovery rate was 54%. Bronchoalveolar lavage fluid (BALF) showed an increased cell count of 3.1 × 10^5^/mL, an increased lymphocyte fraction of 25.5%, and a decreased CD4/CD8 ratio of 0.79. Bacteriological examination showed no significant findings of general bacteria or mycobacterium. Blood tests revealed that KL-6 and lactate dehydrogenase (LDH) were within the normal range, and C-reactive protein (CRP) was mildly elevated. Carcinoembryonic antigen (CEA), a tumor marker, was not elevated. Conversely, anti-ARS antibodies were positive. Serum creatine kinase (CK), aldolase, and anti-Jo-1 antibodies were normal, but anti-PL-12 and anti-Ro-52 antibodies (EUROLINE Myositis Profile 3) were positive (Fig. 2). There were no collagen-related symptoms, such as muscle weakness or skin findings, and the clinical symptoms were only fever and cough.

**Fig. 3:**
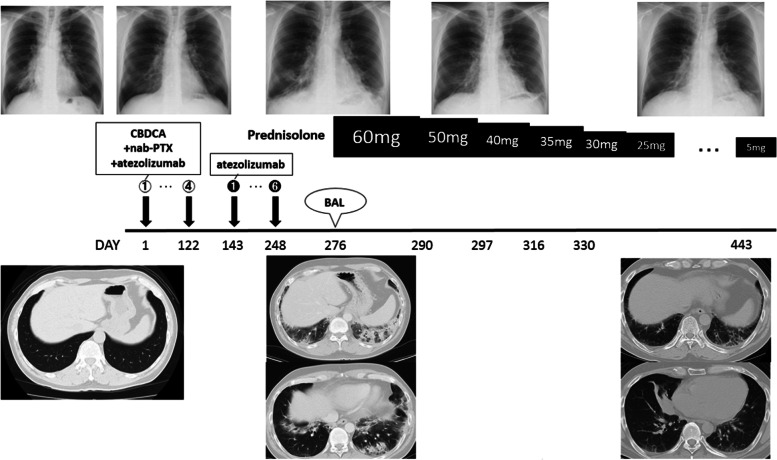
The patient’s clinical course after atezolizumab administration

**Fig. 4:**
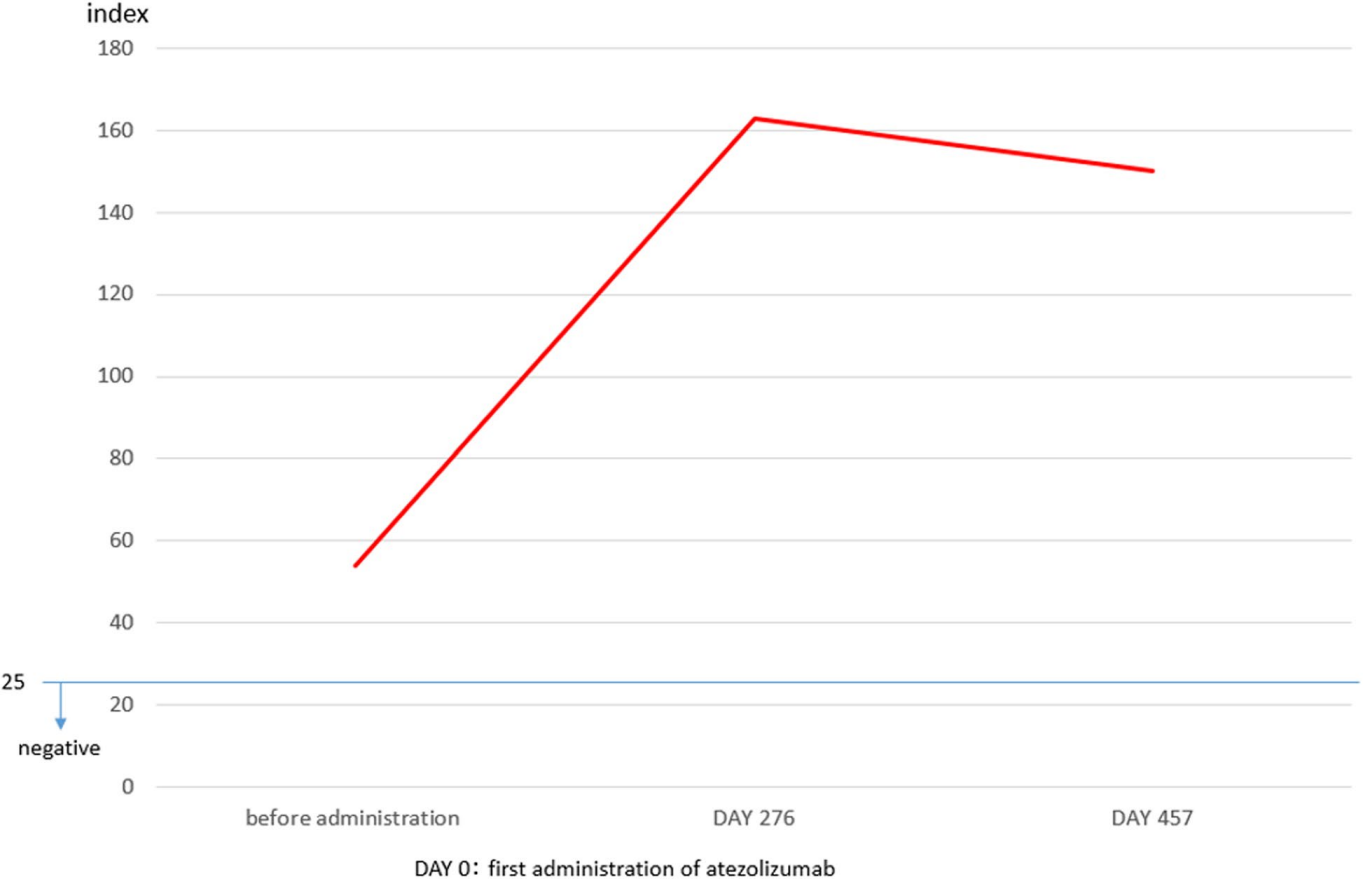
Progress of anti-aminoacyl-tRNA synthetase (ARS) antibody titers

Based on these results, we diagnosed the illness as ASS-ILD and started treatment with prednisolone (PSL) 60 mg/day (1 mg/kg/day). On the second day of treatment with prednisolone, the patient’s fever resolved to normal, and his coughing symptoms showed remission. Chest X-ray showed improvement of the shadows in the bilateral lower lung fields. The PSL treatment was gradually decreased, and the patient is currently receiving 5 mg/day PSL with no worsening of the ILD (Fig. 3). During the course of treatment, anti-ARS antibodies were measured from the stored serum before atezolizumab administration and were positive (index 53.8), and an increase in anti-ARS antibody titers was observed after atezolizumab administration (Fig. 4). However, there was no pneumonia or myositis at the time of serum collection prior to atezolizumab administration. The patient's lung cancer course was followed by the discontinuation of atezolizumab; a PR has been maintained ever since, and the patient will continue to be followed as an outpatient.

## Discussion and conclusion

This patient developed ILD during maintenance therapy with atezolizumab and was subsequently found to be positive for anti-ARS antibodies. ARS, a cytoplasmic antigen, is an enzyme that synthesizes aminoacyl-tRNA by binding specific amino acids to the corresponding tRNA. Anti-ARS antibodies are specific to patients with polymyositis and dermatomyositis [9]. Among anti-ARS antibodies, it has been reported that anti-PL-12 antibody-positive ASS has few muscular symptoms and is mainly associated with pulmonary disease, and this case had only interstitial lung disease [10]. On CT, patients with myositis-related ILD such as ASS-ILD often have radiological traction bronchiectasis, consolidation and volume loss predominantly in the lower lung regions, with nonspecific interstitial pneumonia (NSIP) or NSIP with an organizing pneumonia (OP)/fibrosing OP (FOP) pattern [11–15]. In this case, high-resolution CT showed volume reduction in the lower lobes of both lungs and peribronchial consolidation, characteristic of the FOP pattern. ASS-ILD in general has a high rate of relapse with steroid reduction [2], and the remission rate of anti-PL-12 antibody-positive patients is 5.6%, with a low response rate and poor prognosis [16, 17]. In addition, anti-Ro-52 antibodies are said to occur simultaneously with myositis-related antibodies such as anti-ARS antibodies [18]. Shi et al. reported that rapidly progressive interstitial lung disease develops at a high rate in patients simultaneously positive for anti-ARS and anti-Ro-52 antibodies, and the presence of anti-Ro-52 antibodies may be a prognostic factor in ASS-ILD [19]. Nevertheless, the patient was in remission after the discontinuation of atezolizumab and steroid therapy and remains an outpatient without exacerbation.

The incidence of ILD caused by ICIs administration is estimated to be about 10% [20]. Since the patient developed ILD after receiving atezolizumab, we considered that ICI-induced ASS-ILD was an irAE, and we decided to discuss the relationship between ICIs and ASS-ILD. Since CD4-positive T cells express immune checkpoint molecules such as programmed death-1 (PD-1), we considered the possibility that ICIs induce autoantibody production from CD4-positive T cells via B cells and plasma cells, resulting in the production of new anti-ARS antibodies. We examined the stored serum before atezolizumab administration and found that anti-ARS antibodies were positive even before administration. Therefore, it was ruled out that new antibodies were produced by ICIs. However, anti-ARS antibody titers increased after the administration of atezolizumab compared to before, suggesting that the administration of ICIs increased the level of preexisting autoantibodies [21]. The administration of ICIs to patients with a preexisting autoimmune disease is considered a risk factor for relapse of autoimmunity and the development of other irAEs [22, 23], and most clinical trials exclude such patients. However, there are some reports that flares and irAEs in patients with a preexisting autoimmune disease who receive ICIs are often mild and do not need to interrupt ICIs administration [24–27]. However, these irAEs do not include pneumonitis, and ICIs should be discontinued for grade 2 pneumonitis (CTCAE version 5.0) as usual. The patient in this study had grade 2 pneumonitis, so atezolizumab was discontinued. However, his ILD was relatively mild, and there was no ILD exacerbation with steroid titration. There is a report that once ICI-ILD is overcome, severity grade is not associated with prognosis [20].

From the above, there is a possibility that the ASS-ILD in this patient was caused by an increase in the anti-ARS antibody titer due to atezolizumab administration, which the patient had before receiving ICIs. Assuming, as mentioned earlier, that there are many mild cases of relapse or flare due to ICIs use in preexisting autoimmune disease patients, the results suggest that the discontinuation of ICIs and the administration of steroids may result in a favorable treatment course for newly developed ASS-ILD patients with asymptomatic anti-ARS antibody-positive disease. In addition, the prognosis may be favorable if the ILD is overcome, as is usually the case with ICI-ILDs.

We experienced a case of ASS-ILD as an irAE after treatment with ICIs. The pretreatment serum was examined after treatment for ASS-ILD, and anti-ARS antibodies were positive even before ICIs treatment. We also found that anti-ARS antibody titers were elevated after ICIs treatment compared with those before treatment. ASS-ILD may be caused by an increase in preexisting autoantibodies due to ICIs administration, but there are currently no reports associate antibody titers with the development of ILD. We need to accumulate more cases to determine whether measurement of anti-ARS antibodies prior to ICIs administration is useful in predicting the development of ASS-ILD and the relationship between antibody titers and the development of ILD.

## Data Availability

All data and materials used during the current study are available from the corresponding author on reasonable request.

## Abbreviations

ARS: Anti-aminoacyl-tRNA synthetase
ILD: Interstitial lung disease
ASS: Anti-synthetase syndrome
ICIs: Immune checkpoint inhibitors
irAE: Immune-related adverse events
AUC: Area under the blood concentration–time curve
nab-PTX: Nab-paclitaxel nab-PTX
PD-L1: Programmed death-ligand 1
CT: Computed tomography
PR: Partial response
CTCAE: Common terminology criteria for adverse events
BAL: Bronchoalveolar lavage
BALF: Bronchoalveolar lavage fluid
LDH: Lactate dehydrogenase
CRP: C-reactive protein
CEA: Carcinoembryonic antigen
PSL: Prednisolone
NSIP: Nonspecific interstitial pneumonia
OP: Organizing pneumonia
FOP: Fibrosing organizing pneumonia
